# Book Notices

**Published:** 1883-03-20

**Authors:** 


### *BOOK NOTICES.*

MANUAL OF GYNECOLOGY: By D. Berry Hart, M. D., F. R. C. P. E., Lecturer on Midwifery and Diseases of Women, School of Medicine, Edinburg; late Assistant to the Professor of Midwifery, University of Edinburg; late President of the Royal Medical Society, Etc.; and A. H. Barbour, M. A., B. Sc., M. B., Assistant to the Professor of Midwifery, University of Edinburg; late President of the Royal Medical Society. Vol. 1. with Eight Plates and one hundred and ninety-two wood cuts. New York: William Wood & Co., 1883.

The above work contains 313 large octavo pages, and is beautifully —even fancifully bound. The typography is excellent, the illustrations many and beautiful.

The author states that he has tried to observe the principle "that the Anatomy, Physiology and Pathology of the pelvic organs form the foundation of good Chemical work. As students, we feel the want of a text-book based on this principle and embodying the most recent views from the various literatures," etc. We regard this as an admirable work, and well up with the times in the special department of Gynecology.

UNIVERSALISM AGAINST ITSELF—Revised Edition. A Scriptural Analysis of the Doctrine. By A. Welford Hall, Ph. D. New York: 23 Park Row. Hall & Co. 1883.

This we regard the ablest and most thorough refutation of the doctrine of Universalism which has ever been published. Every text and every possible argument which has been urged on behalf of the doctrine of Universal Salvation has been successfully met. Upon one point only we cannot go with the author, and that is his position touching the foreknowledge of God, in which he holds that God has and exercises the power of withholding His knowledge of future events, or chooses not to foreknow all things; yet we concede that he makes this point more plausable than at first thought seemed possible. Upon the whole, the book is a remarkable one, and should be read by all who desire information upon the subject of Universalism. It is truly Universalism against itself. Those who have not been refreshed by reading the author's work called *Problem of Human Life*, will find the chapters in the concluding part of the present work, on "The Immortality of the Soul," not only novel but exceedingly instructive and interesting.

